# Insights from Experiences on Antiplatelet Drugs in Stroke Prevention: A Review

**DOI:** 10.3390/ijerph17165840

**Published:** 2020-08-12

**Authors:** Salvatore Santo Signorelli, Ingrid Platania, Salvatore Davide Tomasello, Marco Mangiafico, Giuliana Barcellona, Domenico Di Raimondo, Agostino Gaudio

**Affiliations:** 1 Department of Clinical and Experimental Medicine, University of Catania, 95123 Catania, Italy; marcomangiafico@hotmail.it (M.M.); giuli352@virgilio.it (G.B.); agostino.gaudio@gmail.com (A.G.); 2 General Medicine Division, University Hospital G. Rodolico, 95123 Catania, Italy; ingridplat@gmail.com; 3 Division of Hemodynamic and Interventional Cardiology, Hospital Cannizzaro, 95123 Catania, Italy; davtomasello@gmail.com; 4 Division of Internal Medicine and Stroke Care, Department of Promoting Health, Maternal-Infant, Excellence and Internal and Specialized Medicine (Promise) G. D’Alessandro, University of Palermo, 90127 Palermo, Italy; domenico.diraimondo@unipa.it

**Keywords:** prevention, stroke, aspirin, clopidogrel, cilostazol, dipyridamole, combined drug therapy

## Abstract

Reduction of hazard risk of cerebral ischemic event (stroke, transient ischemic attack (TIA)) represents the hard point to be achieved from primary or secondary preventive strategy in the best clinical practice. However, results from clinical trials, recommendations, guidelines, systematic review, expert opinions, and meta-analysis debated on the optimal pharmacotherapy to achieve the objective. Aspirin and a number of antiplatelet agents, alone or in combination, have been considered from large trials focused on stroke prevention. The present review summarizes, discusses results from trials, and focuses on the benefits or disadvantages originating from antiplatelet drugs. Sections of the review were organized to show both benefits or consequences from antiplatelet pharmacotherapy. Conclusively, this review provides a potential synopsis on the most appropriate therapeutic approach for stroke prevention in clinical practice.

## 1. Introduction

Ischemic cerebrovascular events represent a significant public health problem due to their high morbidity, mortality, and possible disability. Non-invasive (or mini-invasive) diagnostic tests (e.g., computerized tomography (CT) scan, nuclear magnetic resonance (NMR), angio-CT scan, angio-NMR) improve diagnosis of ischemic occurrence. They are mandatory in the decision to treat patients with anti-thrombotic drugs. However, prevention still plays a pivotal role, aiming to lower the impact of stroke as a health and social issue (primary prevention). On the other hand, stroke recurrence, or extension in patients having recent or less recent strokes, is a dangerous event. Several antiplatelet drugs are helpful in primary and secondary prevention of stroke, as suggested from recent or less recent guidelines for prevention and treatment of stroke. Several articles (clinical analysis, meta-analysis, review) focusing on stroke prevention enlarged our knowledge on the efficacy of single or combined drugs to prevent primary stroke, or to reduce risk of recurrence of new events that could worsen clinical outcomes, or to reduce the mortality. Although numerous evidences have shown that antiplatelet drugs are effective for stroke prevention, the bleeding (cerebral, gastric, bowel, renal, and urinary tract) related to their use remains a significant side effect. The present review was planned to overview the literature concerning antiplatelet agents for stroke prevention and to improve our knowledge about the possible deleterious (or dangerous) effects related to their use.

## 2. Methodology of Literature Search for Review

### 2.1. Data Sources and Search

A literature search strategy was developed by an experienced team by consulting with the medical scientific web platform (MEDLINE). The literature search was performed to include most of the published papers or reviews updated to 2019. The search used a combination of keywords (e.g., stroke, cerebrovascular events, antiplatelet drugs, aspirin, clopidogrel, cilostazol, etc.). Search process results were limited to papers published in English.

### 2.2. Data Extraction

Every participant in the search process extracted relevant information, and other participants verified the accuracy and completeness of the data. Each reviewer made a judgement on whether the reported results from the search process were different from or corroborated by findings from subsequent papers.

### 2.3. Objective of the Review

The present review aims to focus on the benefits or disadvantages derived from single or combined antiplatelet drugs treatment for ischemic stroke prevention. Results from studies about the prevention and reduction of stroke and the risk of bleeding are discussed. Sections of the review are organized to show benefits or consequences derived from single or combined antiplatelet pharmacotherapy. The conclusive synopsis is focused on the most appropriate therapeutic approach for stroke prevention in clinical practice.

## 3. Aspirin

Aspirin, also known as acetylsalicylic acid (ASA), is one of the most widely used medications, with an estimated 40,000 tonnes (44,000 tons) (50 to 120 billion pills) consumed each year. It is on the World Health Organization’s (WHO’s) List of Essential Medicines, which lists the safest and most effective medicines needed in a health system.

Inactivation of platelet cyclooxygenase (COX)-1 by low-dose aspirin leads to long-lasting suppression of thromboxane (TX) A2 production and TXA2-mediated platelet activation and aggregation [1].

This effect is necessary and sufficient to explain aspirin’s unique (among other COX-1 inhibitors) effectiveness in preventing atherothrombosis, as well as a shared (with other antiplatelet agents) potential to cause bleeding [2]. Aspirin represents a cornerstone in the prevention of cardiovascular (CV) events, as it reduces the risk of recurrent events by approximately 18% [3]. Results of prospective clinical trials and subsequent systematic reviews have established in well-accepted guidelines that antiplatelet agents are effective for secondary stroke prevention at both acute and chronic stages [4,5]. Aspirin is the most widely prescribed antiplatelet agent as the mainstay for secondary stroke prevention [6]. However, clinical trials reported conflicting results regarding the efficacy of ASA for primary stroke prevention.

### 3.1. Aspirin in Primary Stroke Prevention

#### 3.1.1. Benefits

ASA therapy for primary cardiovascular prevention has been widely studied in the last three decades. The Physicians’ Health Study is a randomized, double-blind, placebo-controlled trial designed to determine whether low-dose ASA (325 mg every other day) decreased cardiovascular mortality [7]. This study did not find differences between ASA low-dose compared to the placebo group for stroke incidence (Relative Risk (RR) 1.22; 95% confidence interval, 0.93 to 1.60; *p* = 0.15). ASA was associated with an increased hemorrhagic risk showing a limited statistical significance (relative risk 2.14; 95% confidence interval, 0.96 to 4.77; *p* = 0.06) [7]. The Primary Prevention Project (PPP) study had stroke (non-fatal) and transient ischemic attack (TIA) as endpoints. PPP was planned as a controlled, centrally randomized, open-label clinical trial to test ASA (100 mg daily) vs. vitamin E (300 mg daily) in reducing the frequency of major fatal and non-fatal cardiovascular events, without any clinically relevant safety implications. ASA reduced non-fatal stroke, TIA (Relative Risk Reduction (RRR) 23%; *p* = 0.014) tighter to other endpoints (any cardiovascular deaths, non-fatal myocardial infarction, peripheral artery disease, and revascularization procedures) [8]. The Hypertension Optimal Treatment (HOT) was planned as a randomized controlled study. The HOT study included patients assigned to ASA or placebo. Enrolled hypertensive patients were randomly assigned to the target blood pressure therapy [9,10]. ASA did not affect the frequency of stroke. Interestingly the study found that ASA reduced the major cardiovascular events (15%, *p* = 0.03) and myocardial infarction (36%, *p* = 0.002). Results from the study did not show a difference in the cardiovascular mortality for ASA (5%, *p* = 0.65) vs. vitamin E (7%, *p* = 0.36). The Women’s Health Study [11], a two-by-two factorial trial evaluating the risks and benefits of the low-dose ASA (100 mg daily) compared to vitamin E (600 IU) was targeted on primary prevention of stroke. The study showed a 17% reduction in stroke risk in the ASA group compared to the vitamin E group (relative risk, 0.83; 95% confidence interval, 0.69 to 0.99; *p* = 0.04). The ischemic stroke was lowered up to 24% in ASA group (relative risk, 0.76; 95% confidence interval, 0.63 to 0.93; *p* = 0.009). In line with these data, it is noteworthy that a long-term follow-up provided evidence that 100 mg of aspirin every other day may reduce the risk of ischemic cerebral vascular events but does not have differential effects on functional outcomes from stroke [12]. The Japanese Primary Prevention of Atherosclerosis with Aspirin for Diabetes (JPAD) trial planned to evaluate low-dose ASA (100 mg once daily) for the primary prevention of atherosclerotic events in type 2 diabetes patients. A low dose of ASA did not reduce the risk of stroke [13]. Stroke and TIA primary prevention were listed as endpoints. The study enrolled 14,464 Japanese type 2 diabetic (DM2) patients older than 60 years, having at least one of the major vascular risk factors. DM2 patients were randomized to receive either 100 mg of ASA or no drug. The rate of any stroke or TIA was reduced in the ASA group (*p* < 0.037), particularly in patients over 65 years old (34% of reduction). The Japanese Primary Prevention Project (JPPP) is a multicenter, open-label, randomized, parallel-group trial with ≤6.5 years of follow-up [14]. After a 5-year follow-up, ASA significantly reduced the incidence of transient ischemic attack (0.26 [95% CI, 0.16–0.42] for ASA vs. 0.49 [95% CI, 0.35–0.69] for no ASA; HR, 0.57 [95% CI, 0.32–0.99]; *p* = 0 .04) [15]. The three most recent and large studies ASCEND, ARRIVE, and ASPREE were focused on ASA in primary prevention of the most serious vascular events. The studies included the primary prevention of stroke as one of the endpoints. In the ASCEND study [16] (7.4 years of follow-up), DM2 patients were randomly assigned to 100 mg daily of ASA or to placebo. Study endpoints included, as serious vascular events, non-fatal stroke and transient ischemic attack. An 8.5% rate of events was found in the ASA group whilst in the placebo group the rate rose to 9.6% (rate ratio 0.88; 95% confidence interval, 0.79 to 0.97; *p* = 0.01). The study showed a 12% reduction rate of new vascular events, including stroke in the ASA group. The ARRIVE study [17] compared 100 mg daily of ASA to placebo for primary prevention of cardiovascular events. The study enrolled high-risk individuals, but it excluded type 2 diabetes patients. Stroke and transient ischemic attack were considered as endpoints (myocardial infarction, unstable angina, death from cardiovascular causes were also considered). In the intention-to-treat analysis, the rate of stroke did not differ when comparing ASA vs. placebo (hazard ratio 1.12; 95% CI, 0.80–1.55; *p* = 0.507). Otherwise, the incidence of the composite primary outcome of myocardial infarction, stroke, unstable angina, or death from cardiovascular causes did not significantly differ between the two study groups (4.3% in the ASA group, 4.5% in the placebo group; hazard ratio, 0.96; 95% CI, 0.81 to 1.13; *p* = 0.60). In the ASPREE, a 5-year follow-up study, which enrolled ≥70 years old individuals [18], the participants were randomized to receive 100 mg per day of enteric-coated ASA or placebo. Regarding stroke rate, no difference was found between ASA and placebo (hazard ratio, 0.89; 95% CI, 0.71 to 1.11; *p* = NS). The Antithrombotic Trialist’ Collaboration meta-analysis [19] on the prevention of stroke analyzed the results from six primary prevention studies. A total of 95,000 individuals at low risk were considered. Results showed a total of 3554 serious vascular events. Meta-analysis concluded that net effect of the ASA was not significant for prevention of ischemic or hemorrhagic stroke (ischemic 0.20% vs. 0.21% per year, *p* = 0.4; hemorrhagic 0.04% vs. 0.03%, *p* = 0.05; other stroke 0.16% vs. 0.18% per year, *p* = 0.08). Concerning all serious vascular events, the meta-analysis stated a 12% proportional reduction (0.51% ASA vs. 0.57% control per year, *p* = 0.0001). A recent review [20] discussed ASA for primary prevention. The authors concluded that the ASA did not affect cardiovascular mortality and had a modest effect on stroke. Authors conclusively affirmed that ASA could not be suggested routinely to patients with no overt cardiovascular disease. This conclusion is a notable opinion as a take-home message for clinical practice.

#### 3.1.2. Bleeding

The Physicians’ Health Study [7] found an increase of bleeding risk with ASA compared to that with placebo (27% vs. 20.4%, RR 1.22; 95% CI, 1.25–1.40; *p* < 0.0001). A high rate of blood transfusion was also reported. (RR 1.71; 95% CI, 1.09–2.69; *p* = 0.02) [7]. Similarly, in the PPP study, bleeding consequences of ASA use were 1.1% compared to 0.3 (*p* = 0.0008). Four deaths were caused by hemorrhage, three out of the four were in the control group, but just one event was in the ASA group [8]. In the HOT study [9], the fatal bleedings (including cerebral) occurred both in ASA and in placebo groups, however non-fatal major bleeds occurred most frequently in patients receiving the ASA (RR 1.8, *p* < 0.001). Additionally, minor bleedings occurred 1.8 times more frequent in patients receiving ASA. In the Women’s Health Study [11,13], reports of gastrointestinal bleeding and peptic ulcer were confirmed by the follow-up questionnaires. The side effects were more common among women assigned to the ASA group than in those in the placebo group (4.6% vs. 3.8%, RR 1.22; 95% CI, 1.10–1.32; *p* < 0.001). One hundred and twenty-seven gastrointestinal bleedings requiring blood transfusion occurred in the ASA group while ninety-one bleedings occurred in the placebo group (RR 1.40; 95% CI, 1.07 to 1.83; *p* = 0.02). In both groups, self-reported hematuria, easy bruising, and epistaxis were reported; these side effects were only slightly higher in the ASA group. In the JPAD trial [14], the number of hemorrhagic strokes and severe gastrointestinal bleedings did not differ between two groups of patients received the ASA. In the ASCEND study, major bleedings (a composite of intracranial hemorrhage, sight-threatening intra-ocular bleeding, gastrointestinal bleeding, or another serious bleeding) were significantly increased (RR 1.29; 95% CI, 1.09 to 1.52; *p* = 0.003). A higher incidence of gastrointestinal bleeding (62% in the upper gastrointestinal section, 33% in the lower gastrointestinal) was found, although fatal bleeding and the hemorrhagic stroke did not increase [16]. In the ARRIVE study [17], the gastrointestinal bleeding incidence was found to be twice in the ASA group compared to that in the placebo group (hazard ratio, 2.1; 95% CI, 1.36 to 3.28; *p* < 0.001). However, no significant differences were found for fatal bleedings [16]. In the ASPREE study, the risk of major bleeding was higher in the ASA group (HR 1.38; 95% CI, 1.18–1.62; *p* < 0.001). There was an increased risk of upper gastrointestinal bleeding (HR 1.87; 95% CI, 1.32–2.66) and any intracranial bleeding (HR 1.50; 95% CI, 1.11–2.02), but hemorrhagic stroke did not increase [18]. Zheng et al. [21] in a pooled total of 13 randomized trials showed a direct link between the ASA and the increased risk of major bleedings (HR 1.43; 95% CI, 1.30–1.56). Mahmoud et al. [22] in a meta-analysis of 11 trials (follow-up of 6.6 years) reported an increase in major bleedings (RR 1.47; 95% CI, 1.31–1.65; absolute risk increase 0.6%; number needed to harm: 250) and the intracranial hemorrhage (RR 1.33; 95% CI, 1.13–1.58; absolute risk increase 0.1%; number needed to harm: 1000) in ASA patients. The meta-analysis of 15 study trials from Abdelazis et al. [23] reported a 50% increase of major bleedings (RR 1.50; 95% CI, 1.33–1.69; number needed to harm: 222), intracranial bleedings (RR 1.32; 95% CI, 1.12–1.55; number needed to harm: 1000), and gastrointestinal bleedings (RR 1.52; 95% CI, 1.34–1.73; number needed to harm: 385). Table 1 summarizes studies on ASA.

#### 3.1.3. Current Opinions on Aspirin in Primary Prevention

In consideration of the vast amount of evidence, current guidelines on aspirin use for primary cardiovascular disease (CVD) prevention are conflicting. The European Society of Cardiology (ESC) guideline recommends against the use of ASA for primary prevention in individuals without CVD due to increased risk of bleeding [24]. Conversely, the U.S. Preventive Services Task Force (USPSTF) guideline recommends low-dose ASA for primary prevention in adults between 50 and 59 years of age, who have a 10-year risk of CVD ≥10% without an increased risk of bleeding [25]. Although people with diabetes represent a separate population with a twofold increased risk of cardiovascular events, the statements from the two guidelines appear to be controversial. In patients with diabetes at high/very high risk, ESC guidelines report that aspirin (75–100 mg/day) may be considered in primary prevention in the absence of clear contraindications. In contrast, its use is not recommended in patients at moderate CV risk. The USPSTF advocates low-dose aspirin in adults aged 50–59 years with 10-year cardiovascular risk ≥10% and not at increased risk of bleeding, for primary prevention based on age regardless of the presence or absence of diabetes. The 2018 guidelines from the American Diabetes Association recommend aspirin therapy for primary prevention in those with diabetes and high cardiovascular risk without any susceptibility to bleeding [26]. The review edited by Capodanno et al. [20] does not suggest routine ASA for patients without overt CVD.

### 3.2. Aspirin in Secondary Stroke Prevention

ASA in secondary prevention on stroke was analyzed by Dutch TIA [27] and U.K.TIA [28] trials. They utilized different doses of the drug, 30–283 mg/daily and 300–1200 mg/daily, respectively. Results showed no effect of ASA in the secondary prevention of stroke. Data from a six study meta-analysis showed no difference in stroke reduction when low (<100 mg/daily), medium (300–325 mg/daily), or high (>900 mg/daily) dosage was used. Another meta-analysis of six studies concerning a low dose of aspirin in secondary stroke prevention observed a 25% reduction in the risk of stroke (95% CI, 0.65–0.87) in patients with previous cardiovascular events including ischemic stroke. However, a meta-analysis showed that those patients had several severe bleedings [29].

As a professional message, the American Heart Association/American Stroke Association recommended ASA (75–325 mg daily) in preventing new stroke events in patients with a previous acute cerebrovascular event [30]. Table 2 summarizes studies on ASA in secondary stroke prevention.

## 4. Adenosine Diphosphate P2Y12 Antagonists

The thienopyridines clopidogrel and prasugrel are prodrugs that require liver metabolism to form their active metabolites, which irreversibly bind to P2Y12. After intestinal absorption, clopidogrel is mostly metabolized into inactive metabolites by ubiquitous esterase enzymes. The remaining part (15%) undergoes activation by the hepatic cytochrome P450 (CYP450) enzymatic pathway. Clopidogrel activation requires a two-step of the oxidative conversion process, first to 2-oxo-clopidogrel then to active thiol metabolite. Both steps involve several hepatic CYP isoenzymes. Prasugrel as a prodrug first undergoes rapid de-esterification to intermediate thiolactone and then is converted in the liver to the active metabolite in a single CYP-dependent process. Clopidogrel and prasugrel are irreversible antagonists of the P2Y12 receptor. Ticagrelor is a direct-acting, reversible, noncompetitive antagonist of the P2Y12 receptor and does not need metabolic activation. Active metabolites of the thienopyridine prodrugs bind covalently to the P2Y12 receptor, leading to irreversible, indirect platelet inhibition. There are the newest direct-acting P2Y12 inhibitors (cangrelor, elinogrel) that change the conformation of the P2Y12 receptor [33]. Table 3 summarizes studies on adenosine diphosphate P2Y12 antagonists for the secondary prevention of ischemic stroke.

### 4.1. Clopidogrel

#### 4.1.1. Benefits

Results from studies support clopidogrel use for secondary prevention of non-cardioembolic stroke. The CAPRIE study [34] is the major comparator study focused on efficacy in reducing the risk of clinical thrombotic events. The CAPRIE study is a randomized, blinded, international trial designed to compare clopidogrel (75 mg daily) vs. ASA (325 mg daily) in reducing the risk of a composite outcome cluster including ischemic stroke. No significant differences were found in ischemic stroke reduction between the two drugs. Notably, CAPRIE showed an 8.7% reduction (95% CI, 0.3–16.5) of relative risk for the composite outcome, including ischemic stroke, myocardial infarction, or vascular death. The safety profile of clopidogrel was comparable to that of medium-dose ASA. The effects of clopidogrel plus ASA on stroke prevention were compared to those of Clopidogrel alone in the MATCH study [35] that enrolled patients with ischemic stroke or TIA and an additional “high-risk” disease (prior myocardial infarction, prior stroke, diabetes, angina, symptomatic peripheral artery disease). Ischemic stroke was included as one of the composite primary endpoints (myocardial infarction, vascular death, new hospitalization for acute ischemia). The study protocol randomly assigned patients to clopidogrel (75 mg daily) plus ASA (75 mg daily) or clopidogrel (75 mg daily). Combined drugs did not reduce the risk of the primary vascular events compared to clopidogrel alone (RRR 6.4%; 95% CI, -4.6 to 16.3). The PRoFESS study [36] tested patients with non-cardioembolic ischemic stroke randomized to single clopidogrel (75 mg daily) versus ASA-extended-release dipyridamole (25/200 mg twice daily). Results demonstrated similar benefit and risk but did not show a difference between the two treatments for the recurrent stroke as the primary outcome (9.0% vs. 8.8%, hazard ratio 1.01; 95% CI 0.92–1.11). A review of six randomized controlled studies (CAPRIE, European Stroke Prevention Study 2 (ESPS-2), MATCH, CHARISMA, ESPRIT, and PRoFESS) evaluated the secondary prevention of ischemic stroke by different antiplatelet drugs administered alone or as a combination (ASA alone, ASA plus dipyridamole, clopidogrel alone, ASA plus clopidogrel). Clopidogrel and aspirin/dipyridamole combination for long-term administration had favorable results in secondary prevention after a non-cardioembolic stroke or in reducing the TIA [37,38].

#### 4.1.2. Bleeding

Clopidogrel provoked a moderate lower frequency of gastric or the gastrointestinal bleedings compared to ASA. CAPRIE findings [34] demonstrated intracranial hemorrhage (0.33% vs. 0.47%) and gastrointestinal bleeding (0.52% vs. 0.72%), respectively, for clopidogrel or ASA. The two drugs did not differ in safety. Additionally, rash (0.26% vs. 0.10%), diarrhea (0.23% vs. 0.11%), and gastrointestinal discomfort (0.97% vs. 1.22%) were reported by patients.

Clopidogrel did not show the occurrence of severe neutropenia, unlike another antiplatelet drug (i.e., ticlopidine). Results from large or small studies have focused on such pharmacokinetic questions originating from clopidogrel as well as from ASA. On pharmacokinetic concerns, several patients assigned to clopidogrel were classified as clopidogrel non-responders or clopidogrel resistant. Several potential reasons were considered to explain variability in drug response, such as non-compliance, inconsistent absorption, drug–drug interactions, and genetic variability in CYP isoenzyme activity. Moreover, some frequently co-administered drugs (statins, proton-pump inhibitors, and calcium-channel blockers) could interact with clopidogrel metabolism. However, we want to note that our knowledge is limited by some confounding factors. In conclusion, the clinical significance of interactions is still not confirmed.

### 4.2. Ticagrelor, Prasugrel

The newest drugs ticagrelor and prasugrel are still being evaluated for secondary prevention of stroke, but studies did not reach conclusive data. Both the drugs were used to prevent thrombotic consequences in post-acute myocardial infarction stent implantation. There are a few available studies of these drugs at this moment.

#### 4.2.1. Benefits

In the SOCRATES study (ticagrelor vs. aspirin for prevention of recurrent stroke and cardiovascular events in patients with acute cerebral ischemia), ticagrelor was not found to be superior to aspirin in reducing stroke, myocardial infarction, or death at 90 days, except for patients having ipsilateral extracranial or intracranial stenosis [39]. We need additional studies to demonstrate the role played by ticagrelor for secondary prevention of ischemic stroke of different etiology. The PRASTRO study, a phase I trial (prasugrel vs. clopidogrel), did not show a difference between the two drugs in stroke prevention [40]. Additionally, no safety benefits of low-dose prasugrel compared to clopidogrel have been demonstrated, as indicated by the superimposable frequency of bleedings. Further studies are also needed to elucidate the correct dosage of the drug. A recent meta-analysis showed that ticagrelor is associated with a significant reduction in mortality and recurrent cardiovascular events, as compared to traditional treatment of patients treated for the coronary disease but not for those with the non-coronary atherothrombotic disease [41].

#### 4.2.2. Bleeding

A recent comprehensive meta-analysis of 10 randomized trials evaluated the overall bleeding risk associated with ticagrelor [41]. Drug administration was associated with an increase in significant bleedings. Based on the concomitant relevant risk for bleeding, the administration of ticagrelor or prasugrel may not be justified for patients showing clopidogrel resistance or allergy or for patients without acute coronary syndrome.

## 5. Dipyridamole

Dipyridamole is an antiplatelet agent inhibiting the re-uptake of adenosine diphosphate and platelet phosphodiesterases. The European Stroke Prevention Study 2 (ESPS-2) enrolled patients with recent TIA or ischemic stroke to evaluate reduction of stroke risk allocating patients to ASA alone (50 mg daily), modified-release dipyridamole alone (400 mg daily), the two combined agents, or placebo [42]. Combined aspirin plus dipyridamole reduced stroke risk compared to placebo. The odds ratio of reduced risk for combined therapy was 0.59, compared to 0.79 for aspirin, and 0.81 for extended-release dipyridamole. Dipyridamole plus ASA (200/25 mg twice daily) was also compared to clopidogrel for secondary stroke prevention (75 mg daily). Study findings showed no statistical difference between the two drug protocols [37]. Federal drug administration approved dipyridamole as an adjunctive agent for thromboembolism prophylaxis in patients undergoing cardiac valve replacement and for thallium-nuclear stress testing. Dipyridamole is also used off-label for the prevention of stroke. A combination of aspirin with extended-release dipyridamole was permitted for clinical use including for the stroke prevention as an alternative therapy for patients with intolerable headache. The European Medicines Agency (EMA) indicated dipyridamole as an agent for a coronary diagnostic test, for myocardial perfusion imaging adapted for patients unable to undergo adequate exercise stress, and for the measurement of fractional flow reserve (FFR) of single coronary artery stenosis during invasive coronary angiography when repeated FFR measurements are not anticipated.

## 6. Cilostazol

Cilostazol is a phosphodiesterase III (PDE3) inhibitor. The PDE3s enzymes hydrolyze cyclic guanosine monophosphate (cGMP) and cyclic adenosine monophosphate (cAMP). The PDE3 enzymes are located within the cardiac sarcoplasmic reticulum and in the smooth muscle of arteries and veins. These enzymes play a role in regulating both heart and vascular smooth muscle contractility. Cilostazol acts by inhibiting phosphodiesterase activity and by suppressing cAMP degradation. Inhibition of PDE3 allows a high concentration of cAMP in the platelets and blood vessels. The concentration of cAMP subsequently leads to increased concentrations of the active form of protein kinase A (PKA) directly linked to inhibiting platelet aggregation. High levels of the PKA inactivate myosin light-chain kinase, producing a vasodilating effect on smooth muscle cells. Cilostazol is now considered as an antiplatelet drug and is listed in anti-thrombotic pharmacotherapy [43].

### 6.1. Benefits

Cilostazol is suggested to treat patients with peripheral artery disease based on demonstrated clinical efficacy for intermittent claudication. Controlled trials have demonstrated the effectiveness of cilostazol in preventing cerebral infarction. The CSPS study [44] compared cilostazol to placebo in over 1000 Japanese patients and showed that cilostazol reduced the recurrence of cerebral infarction (41.7%, 95% CI, 9.2 to 62.5; *p* < 0.015). The study found a significant reduction in stroke risk for the cilostazol group of patients. On the contrary, findings from the Chinese study, CASISP [45], demonstrated lower reduction of the composite endpoint (including any stroke, ischemic, or hemorrhagic) in the cilostazol group compared to that of the ASA group. The difference did not reach statistical significance. The CSPS2 trial [46] enrolled 2757 Japanese patients with a recent non-cardioembolic cerebral infarction randomized to cilostazol (100 mg twice daily) or ASA (81 mg daily). Results confirmed that cilostazol was not inferior to ASA for recurrent stroke (rates per year of recurrent ischemic or hemorrhagic stroke was 2.7% in cilostazol and 3.7% ASA, respectively: HR 0.74; 95% CI, 0.56–0.98). The Meta-Analysis of Cilostazol versus ASA for the Secondary Prevention of Stroke analyzed the CASISP, CSPS2, GUO 2009, and CAIST studies [47]. Authors concluded that cilostazol was able to reduce the incidence of stroke, with low bleeding risk. Additionally, another updated systematic review and meta-analysis on cilostazol indicated that it is a more effective and safer treatment than ASA. These data support the safety and efficacy of cilostazol for secondary stroke prevention in Asian populations. However, there are as yet no high-quality data regarding the use of cilostazol for secondary stroke prevention in non-Asian ethnic groups. Moreover, the lower tolerability and higher cost of cilostazol compared with ASA may limit its more widespread use for stroke prevention.

### 6.2. Bleeding

Cilostazol compared to ASA is associated with a 73% reduction in hemorrhagic stroke (RR 0.27; 95% CI, 0.13 to 0.54; *p* < 0.0002), and 48% reduction in total hemorrhagic events (RR 0.52; 95% CI, 0.34 to 0.79; *p* < 0.002). Cilostazol showed the trend to lower the incidence of gastrointestinal bleeding (RR 0.60; 95% CI, 0.34 to 1.06; *p* < 0.08). The annual rate of hemorrhagic events (intracerebral hemorrhage, subarachnoid hemorrhage, or other hemorrhage requiring hospitalization) was lower for cilostazol than that for ASA (0.8% versus 1.8%; HR 0.46; 95% CI, 0.30–0.71). Many more patients discontinued cilostazol than ASA (20% vs. 12%). Interesting data on cilostazol in secondary prevention compared to another drug (ASA) were derived from the meta-analysis on the main studies (CASISP and CSP2). Authors evaluated cerebral bleedings (intracranial, extracranial hemorrhage) and gastrointestinal bleeding as separate outcomes. However, only extracranial hemorrhage was found to be significantly higher in the ASA group compared to the cilostazol group [47].

## 7. Combined Drug Therapy

### 7.1. Aspirin Plus Clopidogrel in Secondary Prevention of Ischemic Stroke

#### 7.1.1. Benefits

Risk of recurrence for ischemic stroke during the first week after the TIA is very high and represents an essential therapeutic problem. In patients who are ineligible for thrombolysis therapy, an advanced treatment with ASA and clopidogrel for 21 days and then with clopidogrel alone for 90 days is suggested. The CLAIR [48] and CARESS [49] studies, focused on combined ASA plus clopidogrel therapy, showed a reduction of ischemic stroke without episodes of bleeding. It is of note that both studies enrolled a small sample of patients affected by stenosis of the carotid artery. This therapeutic scheme is considered a valid option, supported by the 2018 guidelines for Management of Acute Ischemic Stroke.

#### 7.1.2. Bleeding

The MATCH [35] study found increased bleeding occurrence for patients assigned to combined ASA plus clopidogrel therapy without a reduction in ischemic stroke rate. The CHARISMA [50] study found an increased risk of bleeding that was not statistically significant. In the POINT study [51], performed in patients with minor ischemic stroke or high-risk TIA, those who received a combination of clopidogrel and aspirin had a lower risk of major ischemic events but a higher risk of major hemorrhage at 90 days than those who received aspirin alone. Results from studies showed that combined ASA plus clopidogrel therapy prevented recurrent stroke but increased the risk of bleeding. Table 4 summarizes studies on aspirin alone or combined with clopidogrel.

### 7.2. Aspirin Plus Dipyridamole

Combined ASA (25 mg twice daily) plus Dipyridamole (200 mg twice daily) therapy was compared to ASA alone (25 mg twice daily) for primary prevention of cardiovascular events. The study included the prevention of ischemic stroke. Findings from the study demonstrated a reduction of ischemic stroke by the combined therapy. The authors did not suggest dipyridamole use for patients having angina, although no correlations were observed between the vasodilatory activity of dipyridamole and myocardial infarction [54]. ESPS-2 [54] and EARLY studies [55] demonstrated the efficacy of combined ASA plus dipyridamole therapy in preventing recurrent ischemic stroke. The meta-analysis by Leonardi-Bee [56] stated efficacy in primary prevention of ischemic stroke with dipyridamole alone or combined with another drug.

### 7.3. Aspirin Plus Cilostazol

Combined ASA plus cilostazol therapy in comparison to ASA alone was examined by a study targeting prevention of the non-cardioembolic ischemic stroke. Results of combined therapy on cerebral worsening were positive both from short-term (fourteen days) and from prolonged therapy (six months) [57]. Another study [58] showed the effect of ASA plus cilostazol in reducing the frequency of ischemic stroke in patients with previous coronary artery stenting. The study did not find an increased risk of bleeding; however, it ameliorated neither the recurrent ischemic stroke nor cognitive capability.

### 7.4. Aspirin Plus Clopidogrel Plus Dipyridamole

The TARDIS study [59] was designed to evaluate the effects of combined triple drugs therapy (ASA plus dipyridamole plus clopidogrel) compared to a two-drug combination (ASA plus dipyridamole) or clopidogrel alone. The study objective was the secondary prevention of ischemic stroke. Results did not show reduced frequency of stroke by triple drugs but raised the risk of significant bleeding. Conclusively, the authors did not suggest triple therapy as a therapeutic option.

## 8. Insights from Experiences

The prevention of cerebral ischemic event (stroke, TIA) represents a cornerstone in clinical practice. Despite results from clinical trials, recommendations, guidelines, systematic review, expert opinions, and meta-analysis, the optimal pharmacotherapy to achieve the objective is still controversial. Aspirin is the oldest, widely studied and most applied drug for stroke prevention. It was recommended by the ESC guidelines and, in low dose, was suggested by the American Task Force for preventive medicine. The American College of Cardiology/American Heart Association (ACC/AHA) did not recommend the use of aspirin, especially in elderly patients and in adults of any age who are at an increased risk of bleeding. The review of the recent large trials (ASCEND, ARRIVE, ASPREE) on aspirin did not suggest aspirin use to patients without overt cardiovascular diseases. Aspirin is the most assigned antiplatelet drug, it showed a small absolute benefit in preventing ischemic stroke and has demonstrated a relative protective effect in older individuals; conversely low-dose aspirin inhibits hemostasis by inducing the A2 thromboxane-dependent function. International guidelines released divergent recommendations and suggestions. To date, there is not a clear consensus on aspirin as an effective antiplatelet drug for primary prevention of arterial diseases. Other antiplatelet drugs were addressed for primary prevention of stroke. Clopidogrel alone or combined with other drugs was considered to be active for secondary stroke prevention. Cilostazol could be considered an exciting option to prevent ischemic stroke, but the data are more convincing for its use for peripheral arterial disease. Dipyridamole combined with aspirin could be suggested in primary prevention.

## Figures and Tables

**Table 1:** Summary of studies on aspirin.

Study	Physicians’ Health Study	Primary Prevention Project	Hypertension Optimal Treatment Study	The Women’s Health Study	Primary Prevention of Atherosclerosis With Aspirin for Diabetes	Japanese Primary Prevention Project	ARRIVE	ASCEND	ASPREE
**Methodology**	randomized, double-blind, placebo-controlled	randomised, open-label	randomized open-label	randomized, double-blind, placebo- controlled	randomized, open-label,	randomized, open-label	randomized, double-blind, placebo controlled,	randomized, double-blind, placebo controlled,	randomized, double-blind, placebo controlled,
**Study Population**	Healthy male	Patients with one or more CAD risk factor	Patients with hypertension	Healthy women	Japanese patients affected by type II diabetes	Japanese patients who were >60 years old and had at least one major vascular risk factor	Patients with moderate risk of CVD	Patients with Diabetes with no evident CAD	Elderly patients with no CVD, dementia, or physical disability
**Patients Enrolled (n°)**	22,071	4495	19,193	39,876	2539	14,464	12,546	15,840	19,114
**Dose**	325 mg od	100 mg od	75 mg od	100 mg od	81−100 mg od	100 mg od	100 mg od	100 mg od	100 mg od
**Results**	44% reduction of MI	consistent reduction in all the endpoints	15% reduction of MACE, and 36% reduction of MI	No difference regarding primary endpoint	No difference regarding primary endpoint	No difference regarding primary endpoint	No difference regarding primary endpoint	12% reduction of serious vascular events rate	No difference regarding primary endpoint
**Relative Risk**	HR 0.56; CI 0.45–0.7; *p* < 0.0001	HR 0.77; CI 0.62–0.96; *p* < 0.001	HR 0.85; CI 0.73–0.99; *p* < 0.03	HR 0.91; CI 0.80–1.03; *p* < 0.13	HR 0.80; CI 0.58–1.10 *p* < 0.016	HR 0.92; CI 0.74–1.160 *p* < 0.5	HR 0.96; CI 0.81–1.13; *p* < 0.60	RR 0.88; CI 0.79–0.97; *p* = 0.01	HR 1.01; CI 0.92–1.11; *p* < 0.79
**Incidence of Stroke**	0.5% vs. 0.4% *p* = NS	0.7% vs. 1.1% *p* = NS	0.4% vs. 0.4% *p* = NS	1.1% vs. 1.4% *p* = 0.04	2.2% vs. 2.5% *p* = NS	2.1% vs. 2.3% *p* = NS	1.2% vs. 1.7% *p* = NS	2.6% vs. 3% *p* = NS	0.3% vs. 0.4% *p* = NS
**Major Bleeding**	27% vs. 20.4% *p* < 0.0001	0.5% vs. 0.1% *p* = NS	3.1% vs. 1.7% *p* < 0.01	4.6% vs. 3.8% *p* < 0.001	1.6% vs. 0.7% *p* < 0.05	Not measured	0.97% vs. 0.46% *p* < 0.001	1.8% vs. 1.3% *p* = NS	1.7% vs. 1.1% *p* = NS
**ICH**	0.1% vs. 0.05% *p* < 0.06	0.04% vs. 0 *p* = NS	0.02% vs. 0.03 *p* = NS	No reported	0.4% vs. 0.2% *p* = NS	0.7% vs. 0.5% *p* = NS	0.13% vs. 0.18% *p* = NS	0.7% vs. 0.6% *p* = NS	0.5% vs. 0.4% *p* = NS

Legend. OD = once a day; HR = hazard ratio; RR = relative risk; CI = confidential interval; NS = not significant; CAD = coronary artery disease; CVD = cardiovascular disease; ICH =intracranial hemorrhage; ARRIVE = A Randomized Trial of Induction Versus Expectant Management; ASCEND = A Study of Cardiovascular Events in Diabetes; ASPREE = ASPirin in Reducing Events in the Elderly.

**Table 2:** Aspirin in secondary stroke prevention.

Study	Methodology	Enrolment	Results	Reference
**DUTCH TIA**	30 mg/day vs. 283 mg/day	3131 patients	>14.7% in 30 mg/day vs. 15.2% in 283 mg/day (HR 0.91, CI 0.76−1.09)	[27]
**UK TIA**	300 mg/day vs. 1200mg/day vs. placebo	2435 patients	>21.6% in 1200 mg/day vs. 22.1% in 300 mg/day vs. 25.1% in placebo (95% CI 0.76−1.09)	[28]
**CAST Trial**	160 mg/day vs. placebo	21106 patients	recurrent ischaemic strokes in aspirin group 167 [1.6%] vs. 215 [2.1%]. 2p = 0.01	[31]
**SALT Study**	75 mg/day vs. placebo	1360 patients	>16−20%	[32]

**Table 3:** Summary of studies with P2Y12 antagonists.

Drug	Class	Administration	Action	Half Life	Loading Dose Maintenance	Drug Interaction	Resistance	Negative Effects
**Clopidogrel**	Thienopyridine	Oral	Liver activation, irreversible inhibition	7–8 h	300−600 mg/75 mg/day	Yes	Yes	Hemorrhage (especially gastrointestinal or intra-cranial), gastro-intestinal upset, peptic ulcer, pancreatitis, rash/pruritus, dizziness, paraesthesia, leukopenia, TTP.
**Prasugrel**	Thienopyridine	Oral	Liver activation, irreversible inhibition	7–8 h	60 mg/10 mg/day	Yes	Yes	Hemorrhage (especially gastrointestinal or intra-cranial), gastro-intestinal upset, peptic ulcer, pancreatitis, rash/pruritus, dizziness, paraesthesia, leukopenia, TTP.
**Ticagrelor**	Cyclopentyl-triazolo-pyrimidine	Oral	Direct, no competitive, reversible inhibition	6–8 h	180 mg/90 mg twice-daily	Not reported	No	Dyspnea, hemorrhage (especially gastrointestinal or intra-cranial), gastro-intestinal upset, gynecomastia in man, bradycardia, mild increase in serum uric acid and serum creatinine.

**Table 4:** Table shows studies performed on ASA with clopidogrel.

Study	Enrolled pts.	Protocol	Results	References
**MATCH**	7599 pts	ASA + CP vs. CP	Recurrent stroke (*p* = 0.353) Major and minor bleeding (*p* < 0.0001 *)	[35]
**CLAIR**	100 pts	CP + ASA vs. ASA	MES in CP + ASA (*p* < 0.025 *) Minor bleeding (*p* > 0.05)	[48]
**CARESS**	230 pts	CP + ASA vs. ASA	MES in CP + ASA (*p* < 0.001 *) Bleeding (*p* > 0.05)	[49]
**CHARISMA**	15,603 pts	ASA + CP vs. ASA	Recurrent stroke (*p* = 0.07) Mild bleeding (*p* < 0.001 *)	[50]
**POINT**	4881 pts	ASA + CP 90 gg vs. ASA	Recurrent stroke (*p* < 0.01 *) Major bleeding (*p* < 0.02 *)	[51]
**CHANCE**	5170 pts	ASA + CP 21 days→CP 90 days vs. ASA	Recurrent stroke (*p* < 0.001 *) Bleeding (*p* < 0.009 *)	[52]
**SPS3**	3020 pts	ASA + CP vs. ASA	Recurrent stroke (*p* = 0.48) Major bleeding (*p* < 0.001 *)	[53]

**Legend**. ASA = aspirin; CP = clopidogrel; pts = patients; MES = microembolic signals on transcranial doppler. * statistical significant.
